# Morphological and mechanical alterations in articular cartilage and subchondral bone during spontaneous hip osteoarthritis in guinea pigs

**DOI:** 10.3389/fbioe.2023.1080241

**Published:** 2023-01-23

**Authors:** Jiazi Gao, Pengling Ren, He Gong

**Affiliations:** ^1^ Department of Engineering Mechanics, Nanling Campus, Jilin University, Changchun, China; ^2^ Department of Radiology, Beijing Friendship Hospital, Capital Medical University, Beijing, China

**Keywords:** hip osteoarthritis, cartilage, subchondral bone, morphology, mechanical properties

## Abstract

**Objectives:** This study aimed to investigate the morphological and mechanical changes in articular cartilage and subchondral bone during spontaneous hip osteoarthritis in guinea pigs.

**Materials and methods:** Hip joints of guinea pigs were investigated at 1, 3, 6, and 9 months of age (hereafter denoted as 1 M, 3 M, 6 M, and 9 M, respectively; *n* = 7 in each group). Morphological and mechanical alterations during spontaneous hip osteoarthritis in guinea pigs were investigated. The alterations included the micromechanical properties of articular cartilage (stiffness and creep deformation), microstructure of the subchondral bone (bone mineral density, bone volume fraction, trabecular thickness, trabecular number, and trabecular separation), micromorphology of the articular cartilage, and surface nanostructure (grain size and roughness) of the articular cartilage and subchondral bone.

**Results:** Micromechanical properties of articular cartilage in 1 M showed the lowest stiffness and highest creep deformation with no significant differences in stiffness or creep deformation amongst 3 M, 6 M, and 9 M. Articular cartilage thickness decreased with age. The earliest degeneration of articular cartilage occurred at 6 months of age, characterised by surface unevenness and evident chondrocytes reduction in micromorphology, as well as increased grain size and decreased roughness in nanostructure. No degeneration at micro- or nanostructure of subchondral bone was observed before 9 months.

**Conclusion:** Morphological degeneration of cartilage occurred before degeneration of mechanical properties. Meanwhile, degeneration of cartilage occurred before degeneration of subchondral bone during hip osteoarthritis. The current study provided novel insights into the structural and micromechanical interaction of hip osteoarthritis, which can serve as a theoretical basis for understanding the formation and progression of osteoarthritis.

## Introduction

Osteoarthritis is a disease of whole joints and characterised by cartilage degradation, joint inflammation, and abnormal bone remodeling in subchondral bone. The occurrence and development of osteoarthritis are closely related to trauma, overload, and aging (Castaño-Betancourt et al., 2013; Richmond et al., 2013; Barr et al., 2015; Glyn-Jones et al., 2015). Osteoarthritis is a complex condition affecting whole joints, in which degenerations of cartilage and subchondral bone play a pivotal role (Wang et al., 2011; Glyn-Jones et al., 2015; Cucchiarini et al., 2016).

Changes in cartilage morphology and mechanical properties can be used to evaluate the progression of osteoarthritis (Franz et al., 2001; Kraus et al., 2010; Rutgers et al., 2010; Iijima et al., 2014; Zuo et al., 2016; Gatti et al., 2022). Ongoing cartilage destruction may lead to progressive loss in joint function. The characteristic features of articular cartilage degeneration include phenotypic changes in cells, chondrocyte hypertrophy, apoptosis, and progressive fibrillation (Aizah et al., 2019; Harlaar et al., 2022; Salucci et al., 2022). Cartilage covering the whole joint surface is the tissue that directly bears the load. Mechanical failure of articular cartilage may lead to wear of the articular surfaces, pain, and eventual loss of joint function. The cartilage elastic modulus reportedly declines with osteoarthritis progression (Wilusz et al., 2013; Harlaar et al., 2022). The reduction in cartilage compressive stiffness is considered to be one of the first detectable signs of articular cartilage degeneration, that is, changes in mechanical properties occur prior to alterations in the composition of the cartilage matrix (Franz et al., 2001). Thus, evaluating the mechanical properties of the cartilage is important to investigate the degeneration process of osteoarthritis (Iijima et al., 2014; Zuo et al., 2016). Limited by small sample sizes and sample thicknesses, indentation technique is considered to be adequate to characterise the stiffness variation in the articular cartilage for the quantitative assessment of early articular cartilage degeneration (Franz et al., 2001).

Although osteoarthritis was once considered a primary disorder of articular cartilage, the subchondral bone structure is now generally accepted to play an important role in the pathological changes of osteoarthritis. Subchondral bone is particularly associated with cartilage degeneration and is thus a tissue of great interest in the investigation of osteoarthritis. The microstructure of subchondral bone determines its capacity to absorb, distribute, and transfer mechanical loading (Huebner et al., 2002; Barr et al., 2015; Zuo et al., 2016; Finnilä et al., 2017; Peters et al., 2018). Abnormal bone remodeling could be observed during the degeneration process of osteoarthritis. The subchondral bone shows thicker subchondral plate, increased bone density and trabecular bone volume fraction, and decreased trabecular separation and structural model index (SMI) with aggravation of osteoarthritis (Carlson et al., 1996; Ding et al., 2006; Wang et al., 2013; Finnilä et al., 2017), which are considered to result from abnormal mechanoregulated bone adaptation (Iijima et al., 2014).

Osteoarthritis alters the structures of joints at the macro- and microscales and influences the nanostructures of subchondral bone and cartilage. To determine the surface nanostructure of biological tissue (such as grain size and roughness), the use of atomic force microscopy (AFM) is feasible because of its ultrahigh spatial resolution, fine force sensitivity, and versatility under various conditions (Dufrêne, 2002; Hsieh et al., 2008; Stolz et al., 2009; Darling et al., 2010; Wen et al., 2012; Ghosh et al., 2013; Han et al., 2017; Danalache et al., 2019; Plaut et al., 2019). AFM could be used to investigate changes in the morphological properties of chondrocytes and collagen fibrils, as well as topographical variations that may occur in osteoarthritis (Hsieh et al., 2008; Wen et al., 2012), thereby providing a reliable basis for in-depth studies on the osteoarthritis process.

Numerous studies have investigated the mechanical properties and morphology of cartilage and subchondral bone for knee osteoarthritis in different ways, but few studies have been conducted on hip osteoarthritis (Li et al., 2013; Finnilä et al., 2017; Fang et al., 2018; Aizah et al., 2019; Gatti et al., 2022). The hip is the largest weight-bearing joint of human body. Hip osteoarthritis is a disease of whole joints and considered to be one of the main causes of chronic pain and disability in the elderly (Castaño-Betancourt et al., 2013). Investigations on morphological and biochemical changes in hip during the process of osteoarthritis, as well as the relationships between cartilage degeneration and subchondral bone degeneration, is important to understand the causation of osteoarthritis and to select targets for osteoarthritis prevention and treatment. In the current work, we hypothesize that the morphological and mechanical alterations in cartilage and subchondral bone are the main factors inducing hip osteoarthritis. Accordingly, in the current work, hip joints of guinea pigs at 1, 3, 6, and 9 months were investigated to enable a systematic analysis on the morphological and mechanical alterations of articular cartilage and subchondral bone during naturally occurring osteoarthritis, including the microstructure of subchondral bone, surface nanostructures of cartilage, and subchondral bone, as well as the micromechanical properties of articular cartilage, which may provide a basis for the prevention and treatment of osteoarthritis.

## Materials and methods

### Sample preparation

A total of 28 male Dunkin–Hartley guinea pigs aged 1, 3, 6, and 9 months old (hereafter denoted as 1 M, 3 M, 6 M, and 9 M; seven rats in each age group) were sacrificed *via* intraperitoneal injection of 100 mg/kg pentobarbital. Bilateral proximal femurs were harvested. The right proximal femurs were fixed with 4% paraformaldehyde (PFA) at room temperature for 24 h and then prepared for micro-computed tomography (micro-CT) scanning and morphological analyses. The left proximal femurs were stored at −20 °C prior to micromechanical test and AFM analyses. All procedures were approved by the Ethics Committee of The First Hospital of Jilin University (No. 2020–010).

### Bone mineral density and microstructural analysis of subchondral bone

Radiographic images of the proximal femur were obtained with a high-resolution micro-CT system (Skyscan 1,076, Skyscan, Belgium) and then used to determine the microstructure of subchondral bone and assess the radiographic images of the proximal femur. The spatial resolution for proximal femur scanning was set to 18 μm, and the specimens were scanned at 70 kV and 142 μA with Al 1.0 mm filter. The region of interest of subchondral trabecular bone was selected as the region between the subchondral plate and growth plate. Bone mineral density (BMD), bone volume fraction (BV/TV), trabecular thickness (Tb.Th), trabecular number (Tb.N) and trabecular separation (Tb.Sp) were calculated based on the micro-CT image data sets by using CTAn software (CTAn, Skyscan, Belgium).

### Micromorphological analysis of cartilage

After micro-CT scanning, the right femoral heads were fixed in 4% PFA for 3 days and decalcified in ethylenediaminetetraacetic acid for 2 weeks. The samples were then dehydrated in a series of alcohol baths (80%, 90%, and 100%, each for 12 h) before embedding in paraffin. Serial sagittal sections (6 μm thick) were obtained using a rotary microtome (Leica RM2255, Germany). Safranin O/Fast green staining was performed to assess the articular cartilage micromorphology.

### Micromechanical test of articular cartilage

Samples were thawed for 6 h at room temperature and attached with cyanoacrylate cement to a testing chamber filled with phosphate buffered saline. An electronic universal testing machine (AG-X plus, Shimadzu, Kyoto, Japan) with a 0.5 mm diameter flat-ended indenter was used, and the indentation test of articular cartilage was performed as described by Iijima et al. (Iijima et al., 2014; Iijima et al., 2015; Iijima et al., 2017). A preload of 0.01 N was applied and equilibration was conducted for 100 s, followed by loading at a strain rate of 0.005 mm/s up to 0.1 N, which was maintained for 300 s. Dynamic stiffness (0.01 N load divided by displacement in the load–displacement curve; Iijima et al., 2017) and creep deformation (deformation of the cartilage from before to after the application of the test load for 300 s at 0.1 N) were obtained (Iijima et al., 2014; Iijima et al., 2015; Iijima et al., 2017).

### Nanostructure analysis of articular cartilage and subchondral bone

Proximal femoral bone specimens with a thickness of 2 mm were cut perpendicular to the femoral neck axis. The samples were then dehydrated in a series of alcohol baths (80%, 90%, and 100%, each for 24 h), ultrasonically cleaned in alcohol for 5 min, and naturally dried at room temperature (Ren et al., 2018).

**FIGURE 1:**
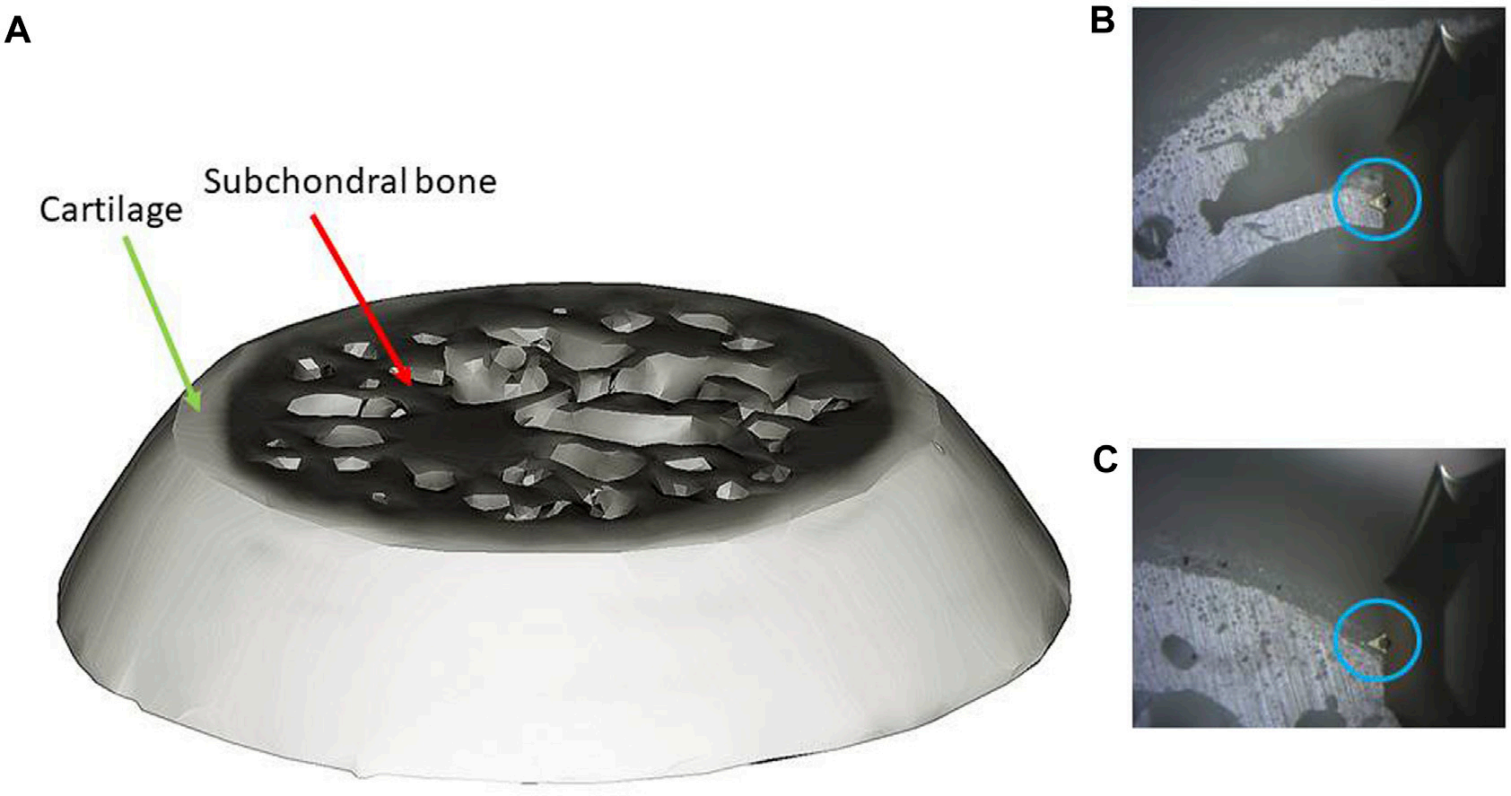
AFM scanning (**(A)**: bone sample; **(B)** scanning positon of subchondral bone; **(C)** scanning positon of cartilage; blue circle: AFM tip).

Each sample was attached horizontally onto the sample disk and imaged with an AFM system (Agilent 5,500, Agilent Technologies, United States). Imaging was performed under ambient conditions in standard AFM tapping mode using a commercial Silicon AFM probe (Tap300AI-G, Budget Sensors Instruments, Bulgaria) with a 125 μm cantilever length, a 40 Nm^−1^ constant force, a 300 kHz resonant frequency, and a tip radius less than 10 nm. Articular cartilage and subchondral bone were scanned and the images were obtained (positions of articular cartilage and subchondral bone for AFM scanning are shown in Figure 1). The size of mineral grains and roughness were measured using NanoScope Analysis version 1.4.0. (Milovanovic et al., 2011).

### Statistical analysis

Data analysis was performed with SPSS 19.0 software (SPSS statistics, IBM Inc., United States). For each group the median and interquartile ranges (IOR, 25th-75th percentile) were calculated. The differences of all the age groups were analysed by the Kruskal–Wallis H test of K independent-sample non-parametric test. Subsequently, Dunn’s multiple comparisons test was performed to determine the differences between groups. The significance level of P was selected as 0.05.

## Results

### BMD and microstructural changes in subchondral bone with age

**FIGURE 2:**
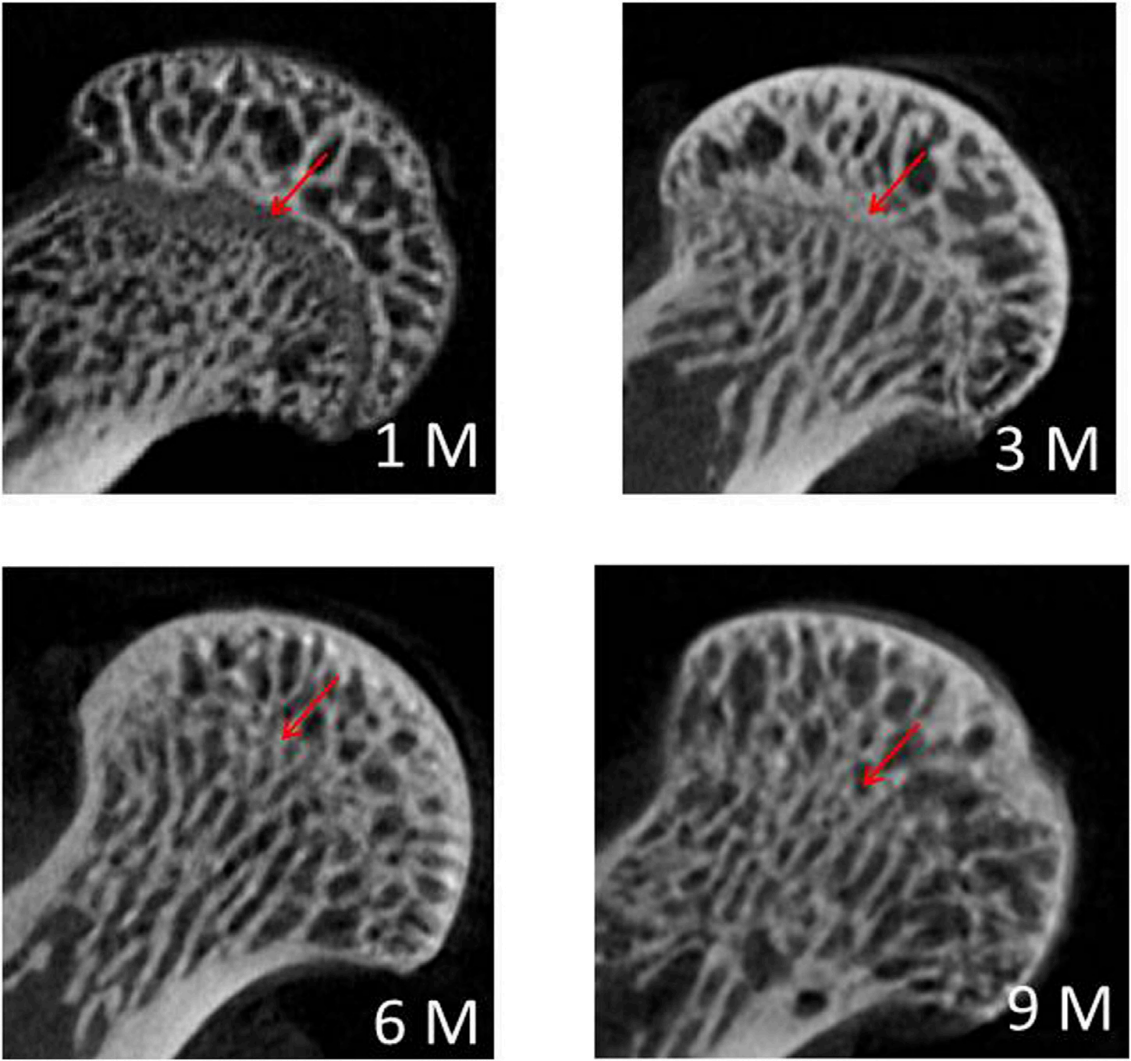
Typical radiographic images of the proximal femurs at 1 M, 3 M, 6 M and 9 M (red arrow: growth plate).

Figure 2 shows the typical radiographic images of the proximal femurs at different months of age. Open growth plates were observed at the femoral heads of 1 M and 3 M. The growth plate was fully closed at 6 M.

BMD and microstructural parameters of the subchondral bone varied obviously with the animal age (Table 1). Increased BMD, BV/TV, and Tb.Th were observed at 3M, 6 M and 9 M with significant difference from 1M (*p* < 0.05). No difference amongst the groups was observed with respect to Tb.N and SMI (*p* > 0.05).

**TABLE 1:** BMD and microstructural parameters of the subchondral bone.

Group (M)	BMD (g/cm^3^)	BV/TV (%)	Tb.Th (mm)	Tb.N (1/mm)	Tb.Sp (mm)
1	0.478 (0.455, 0.497)	39.055 (34.126, 41.816)	0.132 (0.117, 0.139)	3.001 (2.786, 3.126)	0.230 (0.209, 0.238)
3	0.560^a^(0.537, 0.580)	46.817^a^(44.326, 50.818)	0.158^a^(0.153, 0.170)	2.905 (2.828, 2.973)	0.249 (0.237, 0.265)
6	0.635^a^ ^,^ ^b^(0.601, 0.653)	57.530^a^ ^,^ ^b^(53.129, 58.883)	0.186^a^ ^,^ ^b^(0.178, 0.200)	3.028 (2.855, 3.130)	0.249 (0.231, 0.261)
9	0.658^a^ ^,^ ^b^(0.586, 0.690)	60.627^a^ ^,^ ^b^(51.033, 64.423)	0.187^a^ ^,^ ^b^(0.180, 0.205)	3.064 (2.858, 3.216)	0.229 (0.205, 0.259)

Values are presented as median (IQR).

^a^ Significantly different from 1 M; *p* < 0.05.

^b^ Significantly different from 3 M; *p* < 0.05.

### Micromorphological changes in articular cartilage with age

**FIGURE 3:**
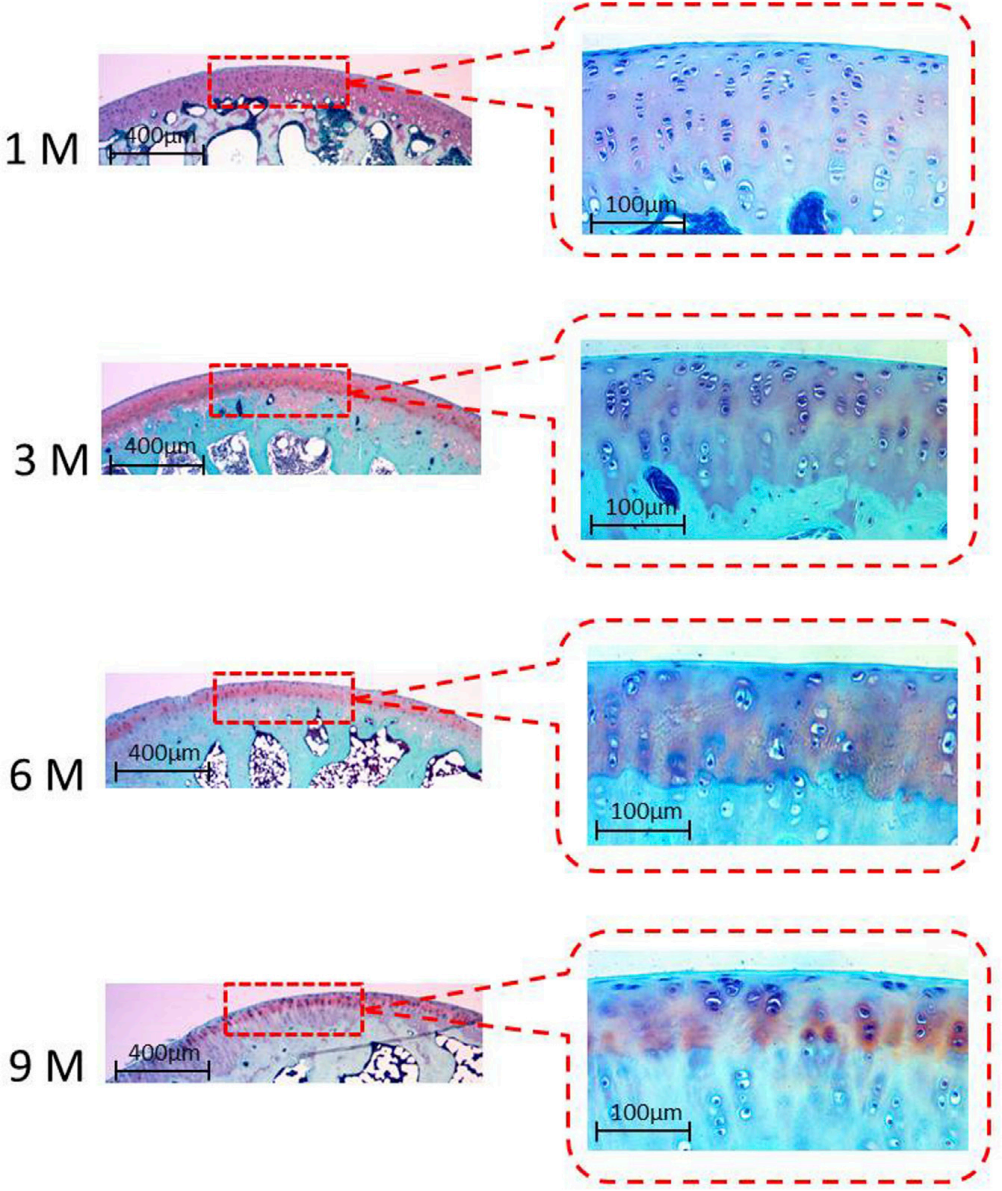
Typical histology images of articular cartilage with Safranin O/Fast green staining at 1 M, 3 M, 6 M and 9M.

Figure 3 shows the typical histology images of articular cartilage with Safranin O/Fast green staining. The thickness of articular cartilage decreased from 1 M to 9 M with the tidal line formed at 3 M. The earliest degeneration of articular cartilage occurred at 6 months of age, characterised by surface unevenness and evident chondrocyte reduction. The degeneration of collagen staining was obvious at 9 months of age.

### Changes in micromechanical properties of articular cartilage with age

**FIGURE 4:**
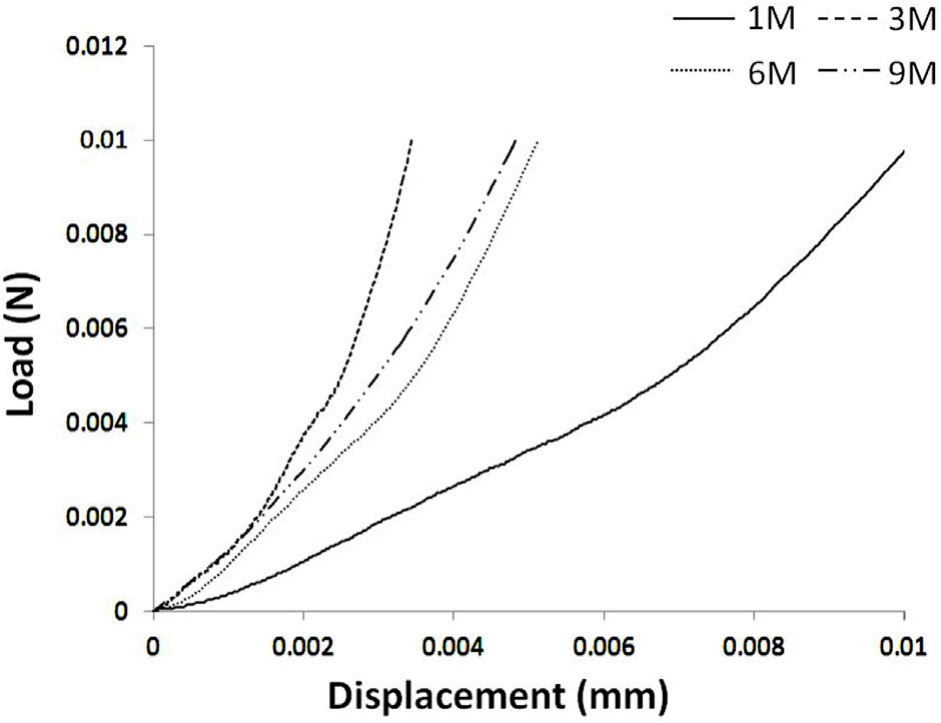
Typical load-displacement curves of articular cartilages at 1 M, 3 M, 6 M and 9 M.

The 1 M group showed the lowest stiffness and the highest creep deformation (*p* < 0.05; Table 2). No significant differences in stiffness or creep deformation amongst 3 M, 6 M and 9 M were observed (*p* > 0.05; Table 2). Typical load–displacement curves at 1 M, 3 M, 6 M, and 9 M are shown in Figure 4, and the articular cartilage of 1 M had the largest displacement under the same load.

**TABLE 2:** Micro-mechanical properties of the articular cartilage at 1 M, 3 M, 6 M and 9 M.

Group (M)	Dynamic stiffness (N/mm)	Creep deformation (µm)
1	1.064 (0.992, 1.320)	18.051 (11.112, 22.601)
3	2.366^a^(2.115, 2.739)	11.323^a^(9.051, 16.020)
6	2.103^a^(1.815, 2.693)	14.255^a^(10.231, 14.661)
9	2.148^a^(1.846, 2.870)	12.184^a^(8.222, 18.317)

Values are presented as median (IQR).

^a^ Significantly different from 1 M; *p* < 0.05.

### Nanostructure changes in articular cartilage and subchondral bone with age

**FIGURE 5:**
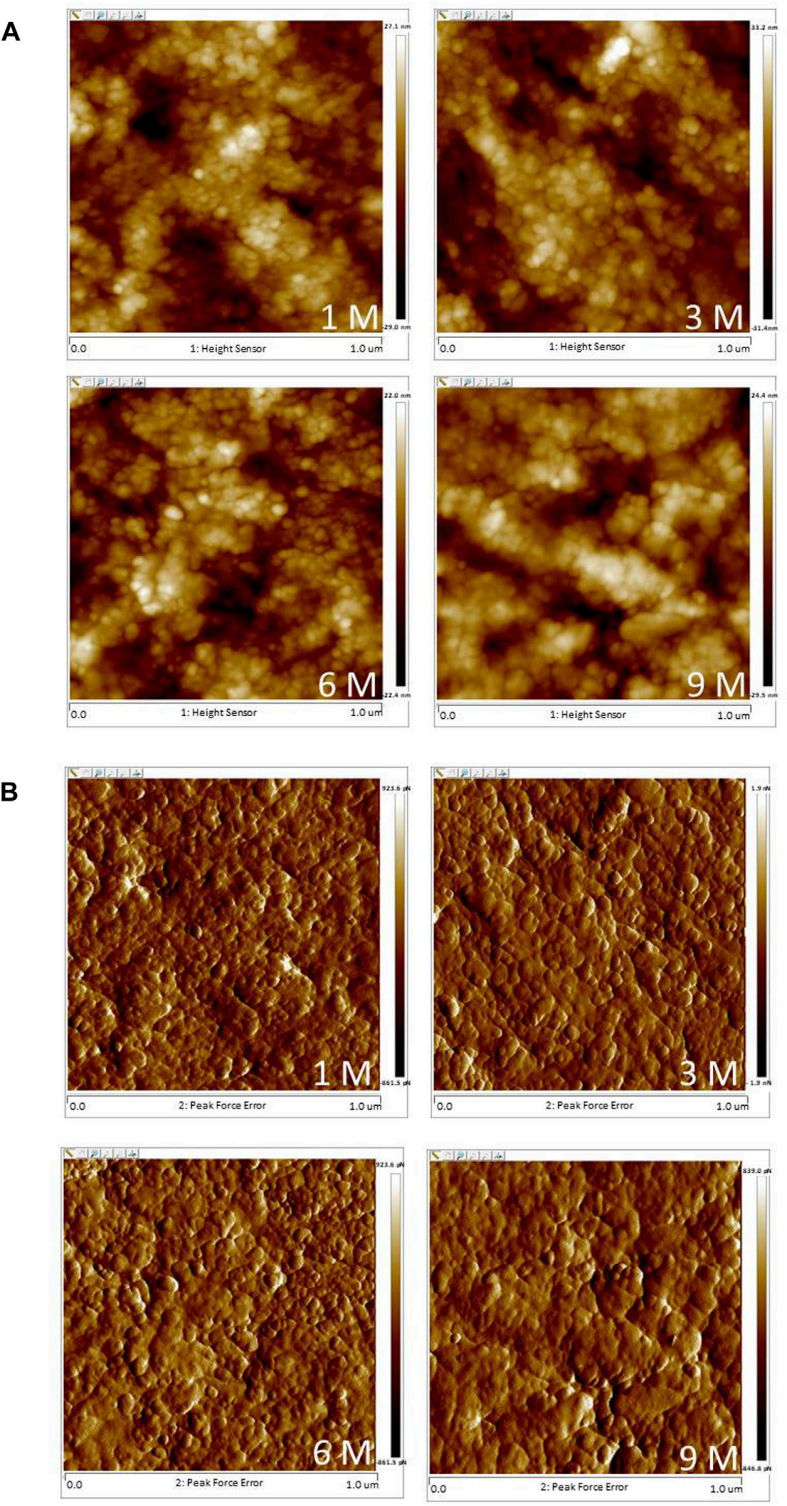
Typical AFM images of the subchondral bone at 1 M, 3 M, 6 M and 9 M (**(A)**: height images, **(B)** phase images; 1 μm × 1 μm scan area).

Typical AFM images of the subchondral bone are shown in Figure 5. In the images, a continuous phase of bone material was found, and obvious mineral crystallinity was observed. Neither grain size nor roughness showed any age-related differences amongst groups (*p* > 0.05; Table 3).

**TABLE 3:** Grain size and roughness from AFM image analysis.

Group (M)	Subchondral bone		Cartilage	
	Grain size (nm)	Roughness (nm)	Grain size (nm)	Roughness (nm)
1	41.588 (37.583, 48.050)	9.360 (6.908, 12.455)	79.946 (73.008, 82.798)	3.931 (2.439, 4.451)
3	37.712 (33.822, 39.776)	8.385 (6.548, 10.545)	58.256 (45.029, 61.277)	4.034 (4.019, 4.980)
6	42.151 (39.339, 44.269)	9.815 (7.908, 13.300)	43.197^a^(32.255, 52.005)	4.858^a^(4.154, 5.394)
9	47.370 (42.293, 58.290)	8.620 (6.485, 9.248)	45.536^a^(33.380, 48.348)	5.510^a^(5.454, 6.157)

Values are presented as median (IQR).

^a^ Significantly different from 1 M; *p* < 0.05.

**FIGURE 6:**
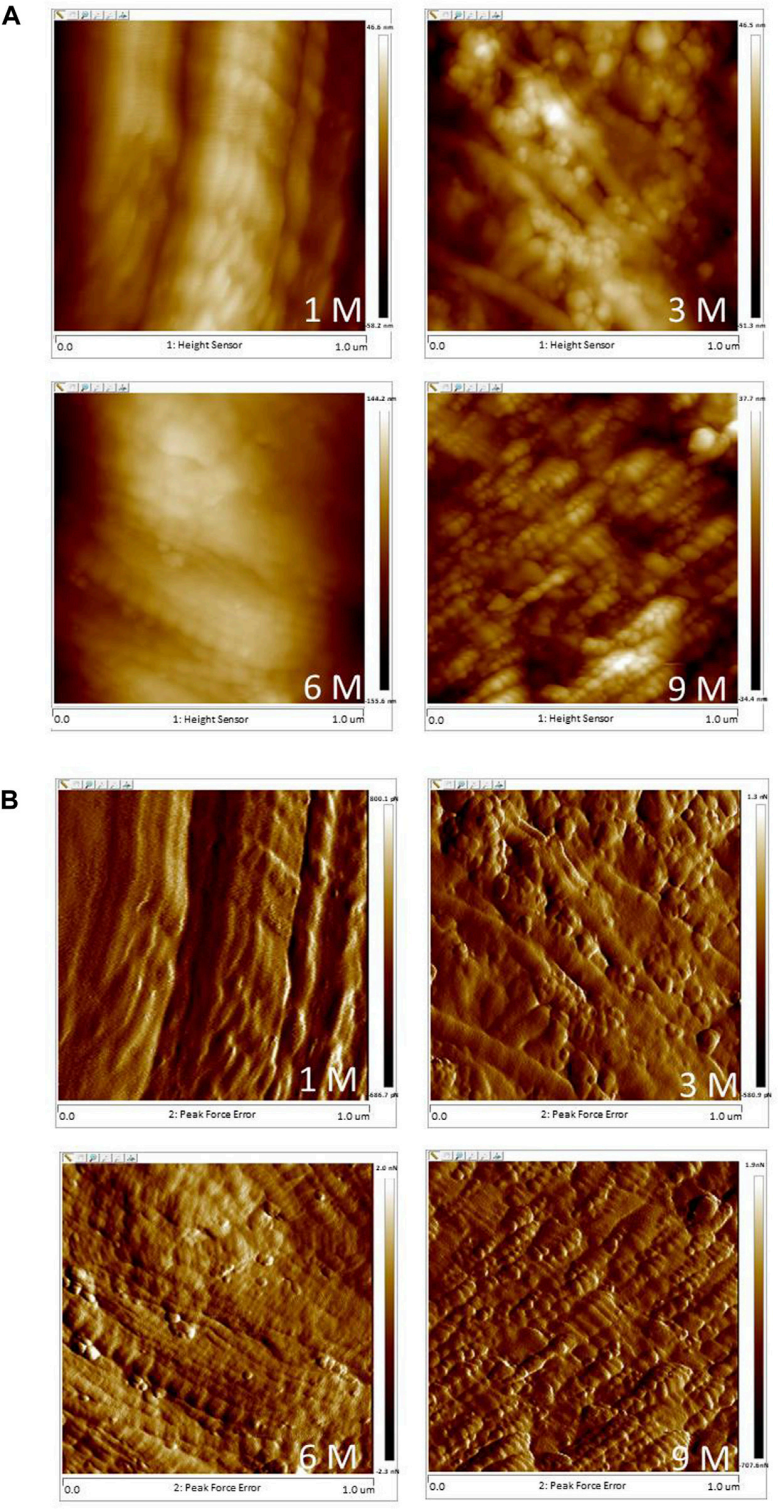
Typical AFM images of the articular cartilage at 1 M, 3 M, 6 M and 9 M (**(A)**: height images, **(B)** phase images; 1 μm × 1 μm scan area).

Figure 6 shows the typical AFM images of the cartilage. Obvious particles were observed on the cartilage surface. More particles could be observed in 6 M and 9 M. The 1 M group showed the highest grain size and the lowest roughness with significant differences from 6 M to 9 M (*p* < 0.05; Table 3). Grain size obviously decreased from 1 M to 3 M (*p* < 0.05; Table 3). The roughness values of 6 M and 9 M were significantly higher than that of 1 M (*p* < 0.05; Table 3).

## Discussion

In this study, hip joints of guinea pigs at 1, 3, 6, and 9 months of age were selected to investigate the morphological and mechanical changes in articular cartilage and subchondral bone during spontaneous hip osteoarthritis. The results obtained in the current study showed that, the morphological and mechanical degenerations of cartilage occurred before those of subchondral bone during the progression of spontaneous hip osteoarthritis. In addition, the morphological degeneration of cartilage occurred before degeneration of mechanical properties.

Osteoarthritis can occur spontaneously in guinea pigs (Kraus et al., 2010; Cucchiarini et al., 2016; Samvelyan et al., 2021). Owing to the strong histological similarities between spontaneous osteoarthritis and human primary osteoarthritis, guinea pigs are commonly used in osteoarthritis studies (Cucchiarini et al., 2016; Samvelyan et al., 2021). In the current study, no obvious characteristics of osteoarthritis were found in the hip joints of guinea pigs at the macro level. However, obvious chondrocyte degeneration and surface structural degeneration related to the development of osteoarthritis were observed at the micro- and nanoscales at 6 months of age, indicating the onset of cartilage degeneration, which were considered to be the early markers of the spontaneous osteoarthritis.

Osteoarthritis is far from being a static disease. It has very distinct characteristics during the different stages of disease progression. Subchondral bone is generally believed to play a substantial role in the degeneration process of osteoarthritis; nevertheless, whether the initial change in osteoarthritis occurs first in bone or in articular cartilage is still controversial. Numerous studies have suggested that subchondral bone changes occur before any signs of degradation in the overlying cartilage emerge. Such changes are characterised by subchondral bone nanostructure changes and acceleration of bone turnover (Radin and Rose, 1986; Li et al., 2013; Wang et al., 2013; Fang et al., 2018; Aizah et al., 2019). However, many researchers still believe that bone changes occur following the degeneration of articular cartilage, or at least at the same time as the degradation of articular cartilage (Li et al., 2013; Finnilä et al., 2017). In the current study, the growth plates of the femoral heads were found to be fully closed at 6 M, indicating skeletal maturity and cessation of longitudinal bone growth. Age-related increases in BMD, BV/TV, and Tb.Th were observed from 1 M to 6 M, with no significant difference of BMD or any microstructural parameter observed between 6 M and 9 M, that is, no obvious degeneration of femoral subchondral bone in guinea pigs existed before 9 months of age. Different from the earliest degeneration of articular cartilage on the medial tibial plateau in guinea pigs observed at 3 months of age (Bendele and Hulman, 1988), the earliest degeneration of articular cartilage on the proximal femur occurred at 6 months of age (characterised by unevenness of cartilage surface and evident chondrocytes reduction, as well as increased grain size and decreased roughness in nanostructure) in the present study. Thus, based on the results obtained herein, the degeneration of articular cartilage may have preceded the subchondral-bone changes in hip osteoarthritis in guinea pigs. Cartilage repair may be an available treatment modality for early osteoarthritis. Physical therapy, such as muscle-strengthening exercises (Bartholdy et al., 2017; Yuenyongviwat et al., 2020); aerobic exercises (Iijima et al., 2015; Kabiri et al., 2018); pulsed electromagnetic field (Ciombor et al., 2003; Yang et al., 2021); extracorporeal shock wave therapy (Chen et al., 2020); ultrasound therapy (Dantas et al., 2021); as well as drug therapy, such as Glucosamine and chondroitin (Fransen et al., 2015), may potentially delay or treat cartilage degeneration effectively. Meanwhile, the molecular mechanism of cartilage degeneration should be further investigated (Ni et al., 2021; Sun et al., 2021; Liu et al., 2022) in order to find targeted drugs that can inhibit cartilage degeneration and delay or prevent disease progression.

A previous study has shown that the mechanical properties of tibial articular cartilage are affected by meniscus (cartilage covered by menisci differs in mechanical properties from that uncovered) (Harlaar et al., 2022). Different from tibial articular cartilage, the proximal femoral articular cartilage has a smooth surface and no tissue coverage that affects its mechanical properties. Decreased mechanical properties are considered to be a remarkable sign of articular-cartilage degeneration (Franz et al., 2001; Fang et al., 2018; Harlaar et al., 2022). In the current work, significantly increased stiffness and decreased creep deformation of the proximal femoral articular cartilage were found from 1 M to 3 M (*p* < 0.05), with no significant changes observed from 3 M to 9 M (*p* > 0.05). This finding indicated that the proximal femoral articular cartilage matured at 3 months. Although no significant differences were found, 6 M and 9 M showed lower stiffness than 3 M which may be related to the degeneration of articular cartilage morphology that occurred at 6 months of age.

Based on its capacity of acquiring 3D surface topographical data at sub-micro- and nanoscales, AFM is extensively used to evaluate the surface characterisations of biological tissues at the nanoscale (Dufrêne, 2002; Stolz et al., 2009; Ghosh et al., 2013; Iijima et al., 2015; Iijima et al., 2017; Ren et al., 2018; Plaut et al., 2019). In the current study, nanostructure changes in articular cartilage and subchondral bone were observed by AFM. Roughness and grain size of trabecular bone were closely related to bone remodeling activity. Large roughness and grain size are considered to be due to delayed bone remodeling and can decrease the mechanical properties of bone at the macroscale (Milovanovic et al., 2011; Milovanovic et al., 2012). However, the present work showed no significant changes in grain size and roughness of subchondral bone amongst different groups, i.e., no degeneration (or at least no abnormal bone remodeling) of subchondral bone occurred.

Observation of nanoscale cartilage-surface characterisations can provide new insights into the initiation and progression of osteoarthritis. Articular cartilage changes due to aging and osteoarthritis could be clearly demonstrated in nanostructure before their morphological changes can be observed, and these architectural changes are then extended to the micro- and macroscales to inflict progressive and irreversible structural and functional damages (Stolz et al., 2009). The present study showed that the roughness of articular cartilage surfaces increased continuously from 1 M to 9 M with the degeneration of articular cartilage, which was consistent with previous ones (Ghosh et al., 2013; Wang et al., 2013). Moreover, the presence of particles on the surface of articular cartilage was obvious from 3 M to 9 M. These changes in the micromechanical properties of articular cartilage may be associated with the changes in nanostructure, thereby further confirming the degeneration of articular cartilage.

The current study should be considered in light of two limitations. First, only hip joints were investigated. In guinea pigs, the mechanical properties and morphological changes in knee articular cartilage and subchondral bone during the development of spontaneous osteoarthritis differ from those of the hip joint. Thus, whilst investigating osteoarthritis, in-depth investigation on knee cartilage and subchondral bone during the development of osteoarthritis and comparison with the hip joint are highly significant in elucidating the mechanism of osteoarthritis occurrence and development. Second limitation of this study is that no degeneration of the hip subchondral bone occurred before 9 months of age. Considering the important role of subchondral bone in the occurrence and development of osteoarthritis, further studies on the mechanical properties and morphological changes in subchondral bone in guinea pigs over the age of 9 months are required. Nevertheless, although restricted by these limitations, changes in articular cartilage and subchondral bone of the hip joint in guinea pigs were carefully investigated. Thus, the results may provide insights into the means of preventing and treating hip osteoarthritis.

## Conclusion

Morphological and micromechanical properties changes in hip cartilage and subchondral bone of guinea pigs at 1, 3, 6, and 9 months were investigated. No degeneration of subchondral bone was observed before 9 months. The earliest degeneration of articular cartilage was observed at 6 months of age and characterised by structural changes at the micro- and nanoscales. Micro- and nanostructural degeneration of cartilage in hip joint occurred before the degeneration of mechanical properties. Structural and mechanical degenerations of cartilage occurred before those of subchondral bone during the progression of spontaneous hip osteoarthritis in guinea pigs. Age-related changes in articular cartilage and bone properties can serve as a theoretical basis for further research on osteoarthritis formation and progression.

## Data Availability

The raw data supporting the conclusion of this article will be made available by the authors, without undue reservation.
